# Diagnosis of triple negative breast cancer based on radiomics signatures extracted from preoperative contrast-enhanced chest computed tomography

**DOI:** 10.1186/s12885-020-07053-3

**Published:** 2020-06-22

**Authors:** Qingliang Feng, Qiang Hu, Yan Liu, Tao Yang, Ziyi Yin

**Affiliations:** 1 Department of Radiology, Linyi Central Hospital, Linyi, China; 2 Department of Healthcare, Linyi Central Hospital, Linyi, China; 3 Department of Surgery, Beijing Tiantan Hospital, Capital Medical University, 119 South 4th Ring West Road, Beijing, 100070 China

**Keywords:** Breast cancer, Triple negative breast cancer, Computed tomography, Radiomics, Molecular subtype, Prediction

## Abstract

**Background:**

To explore the diagnostic value of radiomics features of preoperative computed tomography (CT) for triple negative breast cancer (TNBC) for better treatment of patients with breast cancer.

**Methods:**

A total of 890 patients with breast cancer admitted to our hospital from June 2016 to January 2018 were analyzed. They were diagnosed by surgery and pathology to have mass and invasive breast cancer and had contrast-enhanced chest CT examination before operation. 300 patients were randomly selected for the study, including 100 TNBC and 200 non-TNBC (NTNBC) patients. Among them 180 were used in discovery group and 120 were used in validation group. The molecular subtypes of breast cancer in the patients were determined immunohistochemistrially. Radiomics features were extracted from three dimensional CT-images. The LASSO logistic method was used to select image features and calculate radiomics scores. Receiver operating characteristic (ROC) curve analysis was performed to evaluate the diagnostic value of radiomics scores for TNBC.

**Results:**

Five image features were found to be related to TNBC subtype (*P* < 0.001). These image features based-radiomic signatures had good predictive values for TNBC with the areas under ROC curve (AUC) of 0.881 (95% CI: 0.781–0.921) in the discovery group and 0.851 (95% CI: 0.761–0.961) in the validation group, respectively. The sensitivities and specificities were 0.767, and 0.873 in the discovery group and 0.785 and 0.915 in the validation group.

**Conclusions:**

Radiomic signature based on preoperative CT is capable of distinguishing patients with TNBC and NTNBC. It adds additional value for conventional chest contrast-enhanced CT and helps plan the strategy for clinical treatment of the patients.

## Background

Breast cancer is the most frequent malignant tumor in women with the highest mortality. It remains a worldwide public health dilemma, leading to 450,000 deaths each year [1–3]. Although it is curable in ~ 70–80% of patients with early-stage, non-metastatic disease, advanced breast cancer with distant organ metastases is considered incurable with currently available therapies. During the last 20 years, five intrinsic molecular subtypes of breast cancer (Luminal A, Luminal B, human epidermal growth factor receptor 2 (HER2)-enriched, Basal-like and Claudin-low) have been identified as a result of activation of these genes [4]. Studies have shown that for patients with breast cancer at early stage, the subtype is more important to define treatment strategies, determine the therapeutic outcome and prognosis than histopathologic type [5, 6]. As a heterogeneous disease, the biological characteristics and clinical behaviors of breast cancer are different among the several distinct entities [7]. Triple negative breast cancer (TNBC), a special clinical pathological subtype of breast cancer with negative expressions of estrogen receptor (ER), progesterone receptor (PR), and HER-2 [8], accounts for about 10 to 20% of breast cancer. It is the most aggressive subtype of breast cancer [7]. No effective targeted molecular therapy is available for TNBC and its prognosis is generally poor [9]. Clinically, chemotherapy is the only effective treatment for TNBC [10, 11]. Therefore, early diagnosis of TNBC from non-TNBC (NTNBC) patients is important for better planning of therapy strategies and for predicting the response to neoadjuvant chemotherapy (NCT) administered before surgery for breast cancer [12]. At present, molecular subtyping of breast cancer depends mainly on immunohistochemistry analysis, in which biopsy is used to collect tumor tissue sample. This is an invasive method and there are limitations for sampling and analysis [13]. In contrast, imaging is noninvasive and can reflect the overall characteristics of tumor, allowing analysis of difference among subtypes at molecular level and dynamical evaluation of therapeutic outcomes [14, 15].

As a result of implementation of recommendations by the National Comprehensive Cancer Network (NCCN) guidelines, contrast-enhanced chest CT scan has become a part of routine preoperative examination for breast cancer patients in some hospitals. Different from X-ray mammography, ultrasonography and magnetic resonance imaging of breast lesions, the primary purpose of contrast-enhanced chest CT is to assist clinically staging the disease [16]. It is generally believed that CT is not as good as X-ray mammography to show microcalcification nor as accurate as ultrasound to diagnose breast cystic lesions, although it can incidentally detect breast cancer [17]. It is generally not capable to differentiate benign and malignant breast lesions [18]. However, with the development of radiomics [19–21], which can provide a comprehensive quantification of the tumor phenotype by analyzing robustly [10–12] a large set of quantitative data with characterization algorithms [22], it has been become possible to use imaging features for treatment monitoring and outcome prediction in various cancers [23, 24]. Therefore, we speculate that this method can extract information that is inviable to naked eye from routine preoperative contrast-enhanced chest CT scans for molecular subtyping and characterizing biological features of breast cancer. This would provide new tools and additional information from the routine preoperative CT to characterize the lesions, in addition to assist clinical staging of tumors.

In this study, we attempted to extract radiomic features on the images of preoperative contract-enhanced chest CT for diagnosing TNBC without additional radiation exposure and cost. The findings would optimize planning of treatment scheme for patients with breast cancer.

## Materials and methods

### Subject

This retrospective study analyzed patients treated in our hospital from June 2016 to January 2018. Patients were included if they were proven by pathological biopsy or surgery to have mass and invasive breast cancer and underwent routine preoperative contrast-enhanced chest CT for staging the cancer and had complete immunohistochemical data for molecular subtyping. Patients were excluded if the quality of their images was poor for observation and delineation of tumor foci, or their relevant clinical or pathological data were incomplete. Patients with unusual breast cancer were also excluded. This study was approved by the research ethics committee of Capital Medical University. As it was a retrospective study the requirement for informed consent was waived.

### Molecular subtyping by immunohistochemistry

Immunohistochemistry assessments were made for the expression of ER, PR and HER2 as described previously [25, 26]. Breast cancers were classified into luminal A, luminal B, HER2 overexpression and triple negative (TN).

### CT scanning

Contrast-enhanced chest CT scanning was performed using Brilliance iCT256 (Philips Healthcare, Cleveland, OH, USA), using the following parameters: tube voltage 120 kV, effective tube current auto-mA up to 120 mA, gantry rotation speed 0.5 s, detector configuration 16 × 1.5 mm, slice thickness 1 mm, and transverse field of view 600 mm.

The contrast-enhanced CT was performed as described previously [27]. Briefly, prior to the examination the level of blood creatinine was measured to be less than 1.5 mg/dL. Using a power injector (Ulrich CT plus 150, Ulrichmedical, Ulm, Germany), iodinated contrast agent Uhravist (80-100 ml at 370 mg I / ml) was intravenously administered at flow rate of 4 ml /s. CT images of pulmonary arterial and arterial phases were captured 18 s and 35 s after the injection.

### Extraction of image features

Region of interest (ROI) was identified by two board-approved radiologists (with three and seven years of experience in breast imaging diagnosis, respectively.) using itk-SNAP (http://www.itksnap.org/pmwiki/pmwiki.php). Radiation oncologists were mutually blind of each other’s delineations and were given transversal, coronal, and sagittal views simultaneously for performing delineations. ROI was manually delineated on the arterial phase CT images after removing the unclear or non-mass lesions. The ROI included as much as possible the whole tumor while excluding necrosis, calcification and gas shadow to generate volume of interest (VOI) for the tumor. Radiomics analysis was performed using Metlab (Metlab 2014a, Natick, MA, USA), using an adapted version of CERR (Computational Environment for Radiotherapy Research) with in-house developed radiomics image analysis software to extract imaging features [28]. Intraperson agreement was evaluated using VOIs obtained by the first radiologist at an interval of a week, while the interperson agreement assessed using VOIs obtained by the two radiologists at their first extractions. The radiologists were blind to all other clinical and demographic information of patients, and each other’s delineations.

The image features extracted included the first order statistics, morphological features and texture features. For each CT scan, 182 radiomic features were extracted automatically. The first order statistics included energy, entropy, minimum, maximum, average, median, mean absolute deviation, mean square deviation, standard Quasi deviation, skewness, kurtosis, variance and evenness; the morphological features were surface area, perimeter, concavity, voxel quantity, maximum and the texture features consisting of gray level co-occurrence matrix (GLCM), gray level run-length matrix, gray level size zone (GLSZM) and grey tone difference matrix (GLDM) [22]. A support vector machine (SVM) method (kernel = rbf, C = 0.78, gamma =0.00069) based on recursive feature elimination (RFE) was used to select features for predicting TNBC [29]. All the data of the selected features were normalized with z-score normalization in the training dataset. Redundant features that had low discrimination features with variances lower than the threshold were removed.

The least absolute shrinkage and selection operator (LASSO) logistic regression model was used to select image features in VOIs extracted from arterial phase CT images that might convey diagnostic values for TNBC [30]*.* To generate quantitative radiomics score for a patient, the products of the selected features and the corresponding weighted coefficients were added up to constructed a multiple-feature based radiomics score for predicting survival in the study cohorts as previous described [31].

### Statistical analysis

Data were expressed as means ± standard derivation (SD) and were compared using.

Chi square test, independent sample t-test or the Mann-Whitney U test, as appropriate.

The inter- and intra-observer agreements were evaluated using the intraclass correlation coefficient (ICC) between the first and second measurements of the first radiologist and between the first measurements of the first radiologist and measurements of the second radiologist. A ICC of > 0.75 is considered to have good reliability [32].

Receiver operating characteristic (ROC) curve was used to determine the optimal cutoff score. The area under curve (AUC) in ROC curves was used to assess the diagnostic value of radiomics signatures for TNBC. Using standard statistical formulae, we then determined sensitivity, specificity, PPV, NPV, and accuracy, with the 95% confidence interval (95% CI) value calculated for each parameter. Statistical analyses were performed using R software (version: 3.0.1; http: // www. Rproject. ORG), loaded with relevant packages or functions. *p* < 0.05 was considered statistically significant.

## Results

### Demographic and clinical characteristics

Our study cohort contained 890 patients with mass and invasive breast cancer. 300 patients consisting of 100 TNBC and 200 NTNBC were randomly selected according to the case number. The patients were randomly divided into two groups, 180 were in discovery cohort and 120 were in validation cohort. The patients were all female. TNBC patients were aged between 24 and 79 years with a median age of 49.13 years; the age of NTNBC patients was between 26 and 76 years with a median age of 49.95 years. The ages of the two groups were not significantly different. In addition, the compositions of demographic and clinical characteristics such as age, menstrual status, pathological stage and molecular subtype were similar between the discovery and validation groups (Table 1, *p* > 0.05).

**Table 1:** Comparison of baseline clinical and pathological data of breast cancer patients

Clinical feature	Training group	Validation group	t/χ2 value	*P* value
Age (year)	49.13 ± 11.90	49.95 ± 11.05	−0.009	0.993
Menstrual status (n, %)			1.394	0.238
Before	120 (66.7)	72 (60.0)		
After	60 (33.3)	48 (40.0)		
Pathological stage (n, %)			4.197	0.123
I	12 (6.7)	2 (1.7)		
II	88 (48.9)	53 (44.1)		
III	80 (44.42)	64 (54.2)		
Molecular type (n, %)			1.227	0.754
Luminal A	20 (11.1)	11 (9.2)		
Luminal B	55 (30.6)	36 (30.0)		
HER2 overexpression	43 (23.9)	31 (25.8)		
Triple-negative	62 (34.4)	42 (35.0)		

### The inter- and intra-observer agreement

Analysis found that the ICCs between the measurements of the first radiologist were 0.812 to 0.998, and between the measurements of two radiologists were 0.785 and 0.998. Since the consistency was good to excellent, data from the first radiologist were used for subsequent analysis.

### Establishment of radiomic signature

**Fig. 1:**
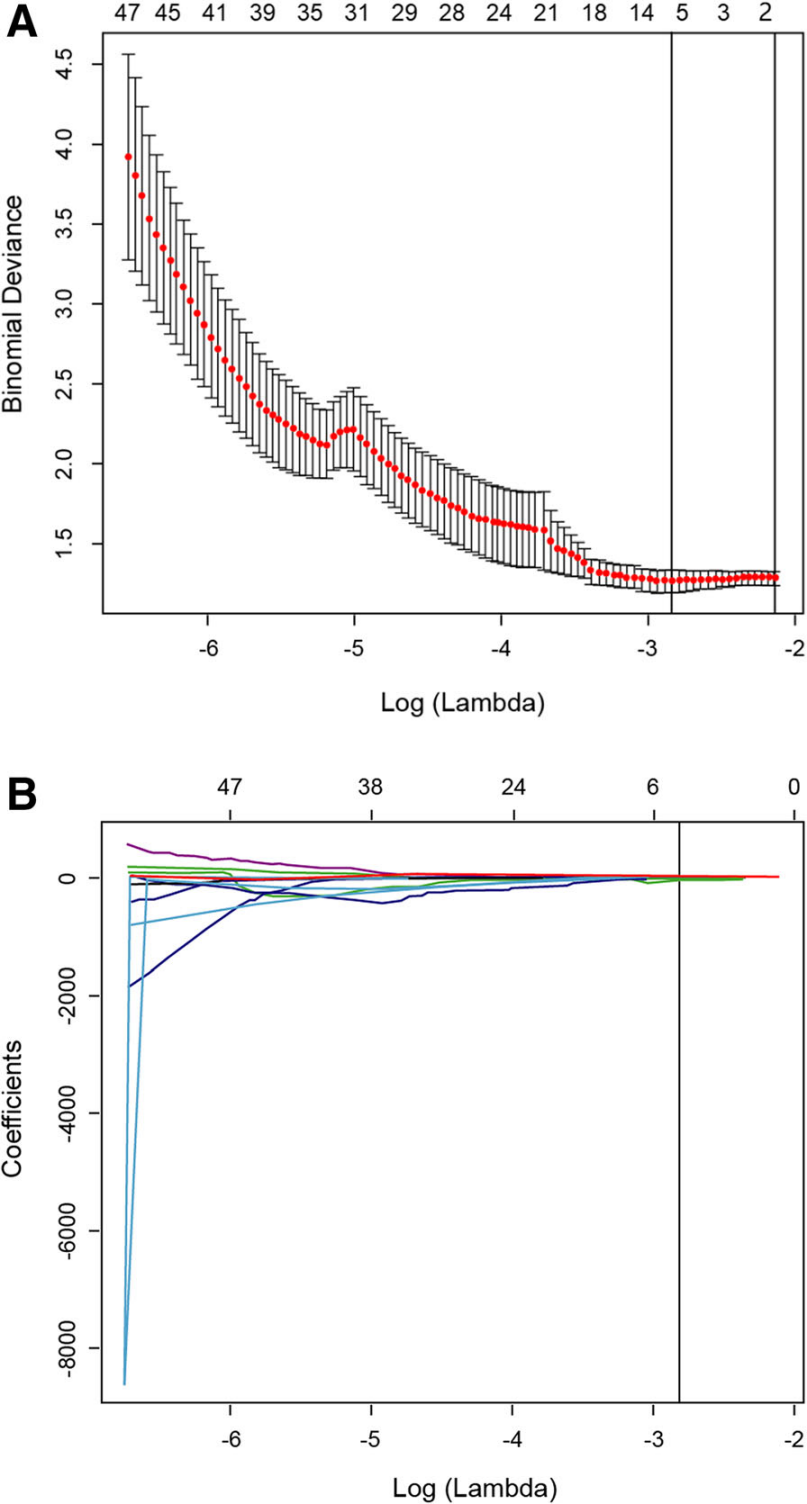
Selection of image features using the LASSO logistic regression models with 10-fold cross validation. **a**. Partial likelihood deviance was plotted versus log (Lambda). The vertical dotted line indicates the lambda value with the minimum error and the largest lambda value where the deviance is within standard error of the minimum. **b**. LASSO coefficient profiles of the features associated with TNBC

Five non-zero image features were identified via the Lasso logistic regression model analysis with 10-fold cross validation (Fig. 1). The scores of radiomic signature were calculated as the sum of selected image features multiplied by their coefficient using formula radiomic score = − 2.21 + 0.03 * compactness – 55.21 * GLZM + 0.18 * GLSZM + 0.36 * band-max + 1.69 * band-mean. In the discovery group, the radiomic score of TNBC group was - 0.426 (interquartile range (*IQR*) - 0.717, − 0.232), which is significantly higher than that of NTNBC group (− 0.824 (*IQR* -1.061, − 0.614), *P* < 0.001). Similarly, in the validation group, the radiomic scores of TNBC and NTNBC groups were − 0.553 (*IQR* - 0.922, − 0.316) and − 0.819 (*IQR* -1.030, − 0.764) with a significant difference between them (*P* < 0.001).

### Assessment of prediction value

**Fig. 2:**
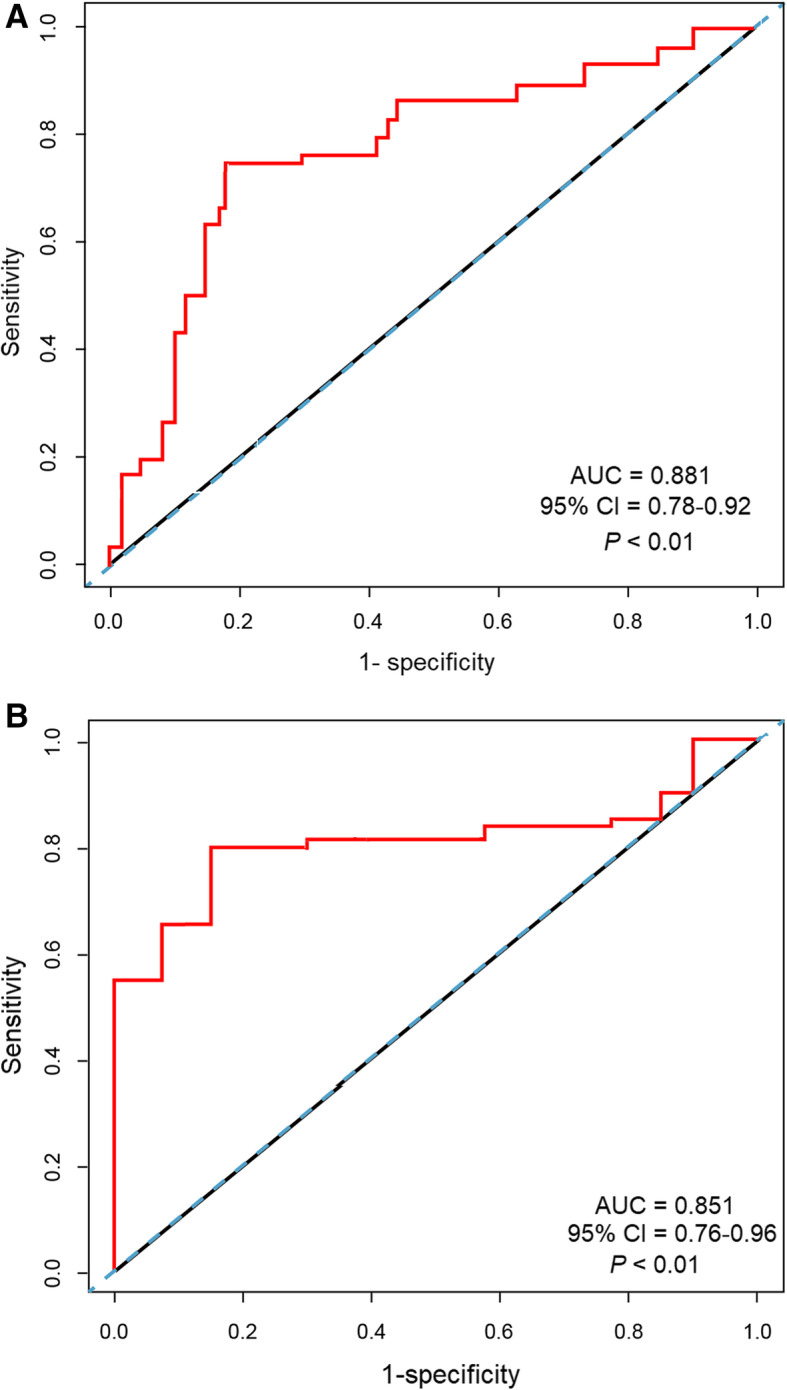
ROC curves of radiomics signatures for discrimination of TNBC in discovery (**a**) and validation (**b**) groups

ROC curve analysis showed that the AUC of radiomic scores to predict TNBC was 0.881 (95% CI: 0.781–0.921) with a sensitivity of 0.767, specificity of 0.873, positive predictive value of 0.755 and negative predictive value of 0.810; correspondingly, AUC in validation group was 0.851 (95% CI: 0.761–0.961) with a sensitivity of 0.785, specificity of 0.915, positive predictive value of 0.850 and negative predictive value of 0.842 (Fig. 2).

## Discussion

Recently, radiomics has provided insights in oncological practice regarding tumor detection, prognosis, subtyping, lymph node metastasis, distant metastasis, and therapeutic response evaluation [30, 33, 34]. In this study, we explored the diagnostic values of radiomics signatures extracted from preoperative contrast-enhanced chest CT to distinguish TNBC and NTNBC. To our knowledge, this is the first report to use CT scans for TNBC prediction. This approach does not generate additional cost and radiation exposure for patients but provide more subjective characteristics from the chest CT for better therapeutic management of patients.

Currently, clinical planning of therapeutic schemes for patients with breast cancer is mainly based on molecular typing of the cancer. The biological characteristics and behaviors of TNBC are different from other types of breast cancer. TNBC patients often response differently and poorly to the same treatment schemes due to the heterogeneity of the tumor [35]. For molecular subtyping, pathological biopsy is needed to obtain tissue samples. However, due to temporal and spatial difference during tumor growth, the biopsy samples may not be representative enough for characterizing tumor tissue. On other hand, image signatures can be quantitatively extracted from the tumor images, and are therefore more subjective and comprehensive. In addition, this approach is non-invasive and quantitative in description of tumor heterogeneity [20, 36, 37].

In our study, five CT image features were identified to have predictive value for TNBC and were used as biomarkers for predicting molecular subtypes of breast cancer. Our results showed that these radiomic signatures are capable to distinguish TNBC and NTNBC.

Previously, a number of studies attempted to use image features extracted mammography and MRI to distinguish TNBC and NTNBC. For example. Agner et al. demonstrated that MRI images contain certain characteristic features that can be captured and quantified for discrimination of triple-negative cancers from non-triple-negative cancers [38]. Ma et al. showed that quantitative radiomic imaging features can be extracted from digital mammograms and they are associated with breast cancer subtypes [39]. Wu et al. found that dynamic contrast enhancement MRI characteristics of breast cancer and background parenchymal enhancement (BPE) may potentially be used to distinguish among molecular subtypes of breast cancer [40]. However, in these studies, the predictions were not cross-validated using dataset from validation cohorts. In this study, the prediction obtained from the discovery group was validated in the validation group, suggesting the prediction is reliable. Using image feature-based radiomic signatures, we showed that the signatures have high specificity in discovery and validation groups (0.873 and 0.915), respectively, for predicting TNBC. Compared with other molecular subtypes, TNBC has poor response to targeted therapy and endocrine therapy, and is more responsive to chemotherapy, which is potentially toxic. Therefore, rationale use of chemotherapy is important to reduce risk. The high specificity of radiomic signatures in predicting TNBC would result in additional value for individualized treatment of breast cancer patients. Since these features are extracted from routine preoperative chest CT, they would not impose additional financial cost and radiation expose to the patients.

Despite our findings, radiomics prediction should be used in connection with preoperative histopathological investigation in order to define the best therapeutic strategy, especially in view of a neoadjuvant systemic treatment, where other clinical (age, comorbidity, etc.) and histopathological (e.g. Ki67 and grading) characteristics should be taken into consideration. In addition, chest CT for staging may be not available for all patients with breast cancer which limits the use of radiomics prediction as described in the work. However, it is likely that the approach can be extended to the pre and post-adjuvant chemotherapy evaluation to identify any characteristics of the residual cell population to the neoadjuvant chemotherapy treatment to guide subsequent surgical and / or radiotherapy treatment based on the cell aggressiveness of the residual cell population.

Although this study provides new approach for diagnosing TNBC, the findings need to be interpreted in the light of several limitations. This was a single-center and retrospective study with limited number of sample. Data extraction was based on existing chest CT scans that were performed for clinical staging of the cancer, which might be not optimal for generating radiomic signatures for detecting the lesions. It is therefore highly desirable to validate our results with large prospective studies and in multiple-center studies.

## Conclusions

Our work shows that radiomic signatures based on image features obtained from preoperative chest CT can be used to distinguish TNBC and NTNBC. This adds clinical values of the routine chest CT and help better planning of therapeutic schemes for the patients.

## Data Availability

The datasets used during the current study are available from the corresponding author on reasonable request.

## Abbreviations

BPE: Background parenchymal enhancement
CT: Computed tomography
TNBC: Triple negative breast cancer
non-TNBC: Non-triple negative breast
ROC: Receiver operating characteristic
AUC: Areas under ROC curve
HER2: Human epidermal growth factor receptor 2
PR: Progesterone receptor
ER: Estrogen receptor
NCT: Neoadjuvant chemotherapy
NCCN: National Comprehensive Cancer Network
ROI: Region of interest
VOI: Volume of interest
GLCM: Gray level co-occurrence matrix
*GLSZM*: Gray level run-length matrix, gray level size zone
GLDM: Grey tone difference matrix
SD: Standard derivation
ICC: Intraclass correlation coefficient
LASSO: The least absolute shrinkage and selection operator
PPV: Positive predictive value
NPV: Negative predictive value
